# Performance analysis of aquaponics system for longifolia lettuce production in different growth media

**DOI:** 10.1038/s41598-025-97095-z

**Published:** 2025-04-23

**Authors:** Atef M. Elsbaay, N. K. Ismail, Mohamed Y. Karawya, Ahmed M. Ragab, Mona M. Kassem

**Affiliations:** 1 Agricultural Engineering Department, Faculty of Agriculture, Kafrelsheikh University, Kafr El-Sheikh, 33516 Egypt; 2 Bio-Eng. Res. Systems Dept., Agric. Eng. Res. Inst. (AEnRI), Agric. Res Center (ARC), Giza, Egypt; 3 Faculty of Fish Resources, Engineering Science Department, Suez University, Suez, Egypt

**Keywords:** Aquaculture, Aquaponics, Nile tilapia, Food safety, Sustainability, Crop quality, Water quality, Fish growth, Lettuce, Ecology, Ecosystem ecology, Freshwater ecology

## Abstract

The aim of the present study on the performance of aquaponics systems is to provide a solution to several sustainability issues and obtain organic food free of chemical contaminants. Where, the water quality for rearing Nile tilapia fish is evaluated in three tanks (T_1_, T_2,_ and T_3_). In addition, assess the best growth media of the lettuce plant (*Lactuca sativa*) in boxes of wood, such as the first box was Crushed Stone (CS), the second box was Crushed Gravel + Crushed Stone (CG + CS), and the third box was Crushed Gravel (CG)) for growing lettuce and also used as a filter to improve water quality. The main experimental work of the present study was carried out during the period from September 2022 to April 2023. The experimental system was designed, fabricated, and implemented in the laboratory at the Al Gamaleya Center (31°51′56.7 N and 31°10′44.2 E), Dakahlia Governorate, Egypt. Fish growth parameters were measured in terms of weight and weight gain weekly, and fish growth indicators and water quality indicators of the system were calculated weekly in the three tanks for 17 weeks, and also lettuce crop quality indicators were measured for the three boxes. For water quality indicators, dissolved oxygen (DO), toxic ammonia (NH_₃_), pH, temperature, total dissolved solids and electrical conductivity were analysed. In addition, lettuce growth parameters were measured in the three boxes with the three different media by leaf number, leaf width, leaf length and root diameter. The effect of fish growth environment (tanks), time, and their interaction as independent variables on water quality indicators were studied. The results indicated that growth rate for fish and lettuce was better in the third tank and third box respectively. However, the current results also showed that the levels recorded in the three tanks for water quality indicators do not exceed the permissible levels required for fish farming and production, but third unit (tank + box) had the best overall result for all parameters under study and where, it was the best in general. The most important results of this analysis were the significant p-values for the indicators NH_3_ and DO with T, and t. The interaction T*t was not significant for DO and significant for NH_3_. The R^2^ values were 0.914 and 0.873 respectively for the growth indicators as follows: NH_3_ and DO respectively.

## Introduction

At limited water availability, environmental pollution, increased fertilizer cost, and depletion of fertile soil, the aquaculture production systems and/or water recycling systems exploit the symbiotic relationship between plants and fish to produce food. This may be one of the good ways to obtain a source of sustainable, safe food. The rationale of integrated agri-aquaculture systems is to take advantage of the resources shared between plant production and aquaculture, such as nutrients and water, to achieve and develop more environmentally and economically viable and more sustainable primary production practices. Aquaculture in Egypt has rapidly expanded, with production increasing from 24,000 Mg (15.4% of total fish production) in 1982 to 1,576,189 Mg (78.7% of total fish production) in 2021 ref.^1^. Aquaculture for food and sustainable development highlights aquaculture as one of the key sectors requiring attention to global food security and sustainable development. The aquaculture industry is one of the fastest-growing sectors in the food industry, supplying more than 50% of the global demand for fish in the face of dwindling natural stocks due to overexploitation^2^. The adoption of Recirculating Aquaculture System (RAS) has significantly advanced aquaculture due to its multiple benefits, including reduced water use through partial reuse of cultured water, decreased environmental impacts from fish farming through improved waste management and nutrient recycling, enhanced sanitation, reduced disease outbreaks, and limited biological control^3^. A recirculating aquaculture system (RAS) is a closed-loop system for fish production in which water is recirculated within the system by maintaining filtration and periodically adding fresh water^4^. Macintyre^5^ stated that the recycling process begins when a product has reached the end of its useful lifetime and would otherwise have been discarded. This idea implies that a circular business model based on the reuse, recycling, or repair of resources and goods should be employed instead of the traditional take-make-waste paradigm. Closed material loops, which imply that resources are utilized further as bulk material, goods, or components, are a requirement for the circular economy concept.

Fish raised in aquaponics systems require a good water quality condition, which means that parameters such as dissolved oxygen, carbon dioxide, ammonia, nitrate, nitrite, and pH must be within acceptable species-specific limits. Sudden changes in the fish stock density, feeding rate, growth rate, or water volume can elicit rapid changes in water quality; therefore, regular measurement of these critical water quality parameters is essential. The deterioration of water quality parameters affects fish physiology, growth rate, and feed efficiency, leading to pathological changes and even mortality under extreme conditions^5,6^. Plants and fish require different conditions for optimum growth regarding pH values for fish survival, which are 6.4-9.0, for nitrifying bacteria (7.0–8.0), and for plants, 6.0–6.5 ref.^7,8^ and water temperature for tilapia was range of 25 to 30 degrees Celsius^9^, dissolved oxygen concentration must be at least above 5 mg/L^8,10^, optimum concentrations of ammonia for tilapia must be less than 0.05 mg/L^11^. Maximum limit of ammonia concentration for aquatic organisms is 0.1 mg /L^12^, acceptable range of total NH_3_-N: less than 4 mg/L and Un-ionized NH_3_-N: less than 0.4 mg/L and the acceptable range of Electrical Conductivity (EC) for freshwater fish generally was 30−5,000 µSiemens/cm^13^.

The nitrogen cycle is predominant in aquaponic systems, which include the conversion of ammonia produced by fish to nitrates, a useful nitrogen source for plants, by bacteria through the process of nitrification. In this way, fish, plants, and bacteria coexist in a balanced common system. Converting residues into resources makes aquaponics a promising and environmentally friendly technique, which, under certain conditions, may allow economic benefits compared to the conventional production systems^14,15^. The plant can be considered as a biofilter for the fish in a mutually beneficial symbiotic relationship by absorbing nutrients from farm waste with the bacteria working to reduce the ammonia through the nitrification process^16,17^. Monsees^18^ showed that the production of lettuce in separate aquaponic systems achieves the same yield and quality as conventional hydroponic systems, but greenhouse gas emissions are drastically reduced due to inorganic fertilizer savings. Another report comparing lettuce production between cultivated in conventional hydroponic and aquaponic solutions of Nile tilapia revealed that the aquaponics solution increased plant growth by about 39% ref.^19^. Lettuce (*Lactuca sativa L.*) is an economically important vegetable and a significant source of essential minerals, as well as vitamins and natural health-promoting phytochemicals, including flavonoids, carotenoids, and L-ascorbic acid^20,21^.

Therefore, the overall objective of this research work is to study the performance of the aquaponics system to provide a solution to several sustainability issues. This was done by identifying the three sub-systems of fish (Nile tilapia), water, and plant growth media and studying some relevant variables. Fish weights were measured, some growth indicators of fish were calculated, water quality indicators were measured, and plant growth indicators (lettuce) were measured. This leads to improving ecosystem health, improving agricultural production, improving aquaculture efficiency, and paving the way for improving system performance and sustainability.

## Materials and methods

### Design and construction of aquaponic system

**Fig. 1:**
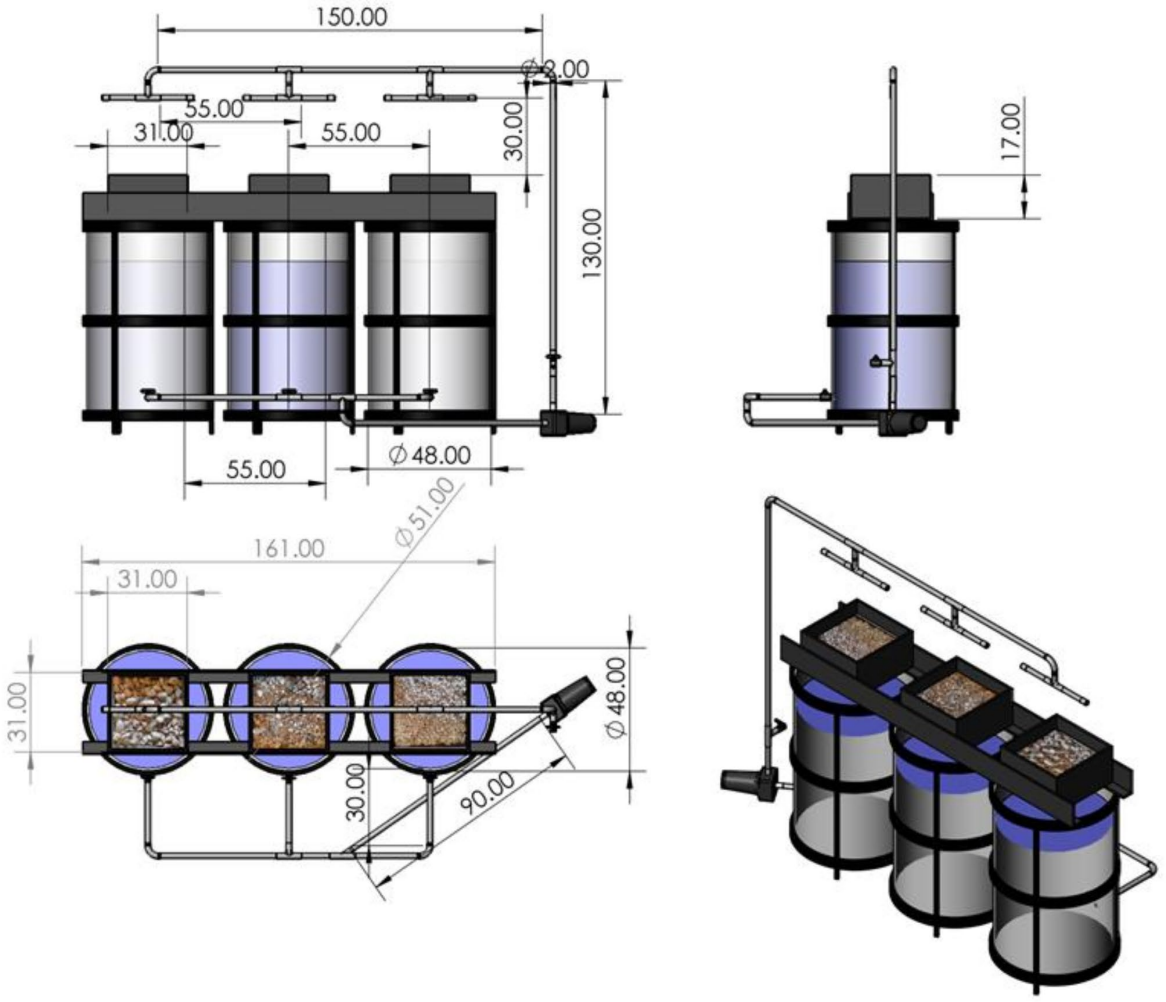
A schematic of diagram for front view, side view, plan view and isometric of the system.

The aquaponic system was designed to consist of three tanks (T_1_, T_2,_ and T_3_) for raising tilapia in the form of a cylindrical cross-section. It is made from transparent acrylic sheets. The external dimensions of each tank were 51 × 73.5 cm. On each tank, a wooden box is placed. The wooden box has the shape of a rectangular prism. It has internal dimensions of 31 × 31 × 17 cm. Different sized rocky media were placed for growing plants in the first box was Crushed Stone (CS), the second box was Crushed Gravel + Crushed Stone (CG + CS), and the third box was Crushed Gravel (CG). The media used to filter the water and increase the concentration of dissolved oxygen in the water and then fertilize the plants with nitrogen through the ammonia dissolved in the water resulting from fish residues. The engineering properties and the calculated measurements of the fish tank and plant boxes are shown in Table 1. A schematic of the diagram for the front view, side view, plan view, and isometric of the system is shown in Fig. 1.

**Table 1:** The geometric properties and measurements calculated for the fish tanks and plant boxes.

Dimensions	Fish tank	Plant box
Outer diameter, cm	51	–
Inner diameter, cm	48	–
Outer height, cm	73.5	17
Inner height, cm	71	–
Length × width, cm	–	31* 31
Height of water/rock, cm	60 cm	10 cm
Total volume of tank/box	128.5 L	0.016337 m^3^
Volume of water/media	108.57 L	0.00961 m^3^
No. of fish/plant	10	3

### Design and construction of irrigation and water recycling system in aquaponic system

**Fig. 2:**
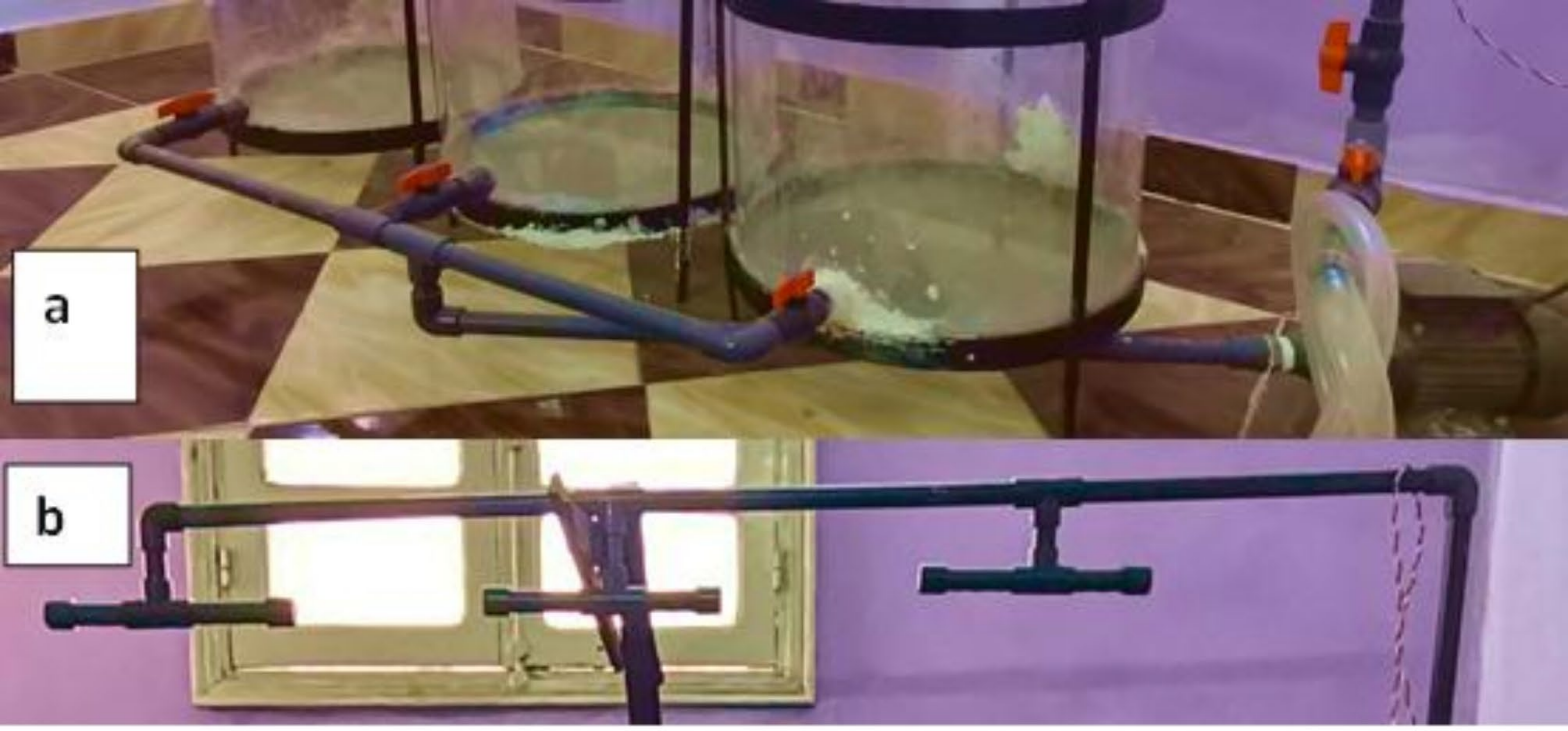
A photograph of the irrigation network (**a**) and water recycling (**b**).

The recycling water network in aquaponic system construction can shows in Fig. 2a and b. It consists of water pump, irrigation tubes and stopcock. The recycle of water were done by suction the water from the tanks using a pump to irrigate the plants every day. Then it returns to the tanks again through the wooden boxes after passing through the plants from which it feeds and filters them. The pump was run of 15 min every hour throughout the experiment from 8.00 AM to 4.00 PM, and this was done using a 24-hour timer. Then an air compressor, an AC 100 W was used during the periods when the irrigation system was stopped supplying the system with oxygen. It blows about 110 *L*/min of air, and this was done using a 24-hour timer.

### Lettuce plants

Seedlings were transplanted when two leaves were completely expanded 30 days after sowing. Seeds of lettuce (*Lactuca Sativa L.*) cv Longifolia, were obtained from the Horticulture Research Institute, Agriculture Research Center, Egypt. Seedlings were set up in the experiment from September 2022 to April 2023. Seedlings were planted in a wooden box with soil from rocks of different sizes. The volume of growth media was 0.00961 m³; there are 3 plants with a cultivation area of 15 × 10 cm².

### Instrumentation and measurements

Water quality was measured weekly using devices during the experiment period. The elements measured are dissolved oxygen using a dissolved oxygen meter, pH by pH meter, ammonia by ammonia MR, and total dissolved solids (TDS), electrical conductivity (EC), and temperature by TDS/EC meter. Fish weight was measured weekly by taking a random sample from the three tanks. Also, some plant parameters were measured weekly, such as number of leaves, leaf length, leaf width, and stem diameter.

### Experimental setup

**Fig. 3:**
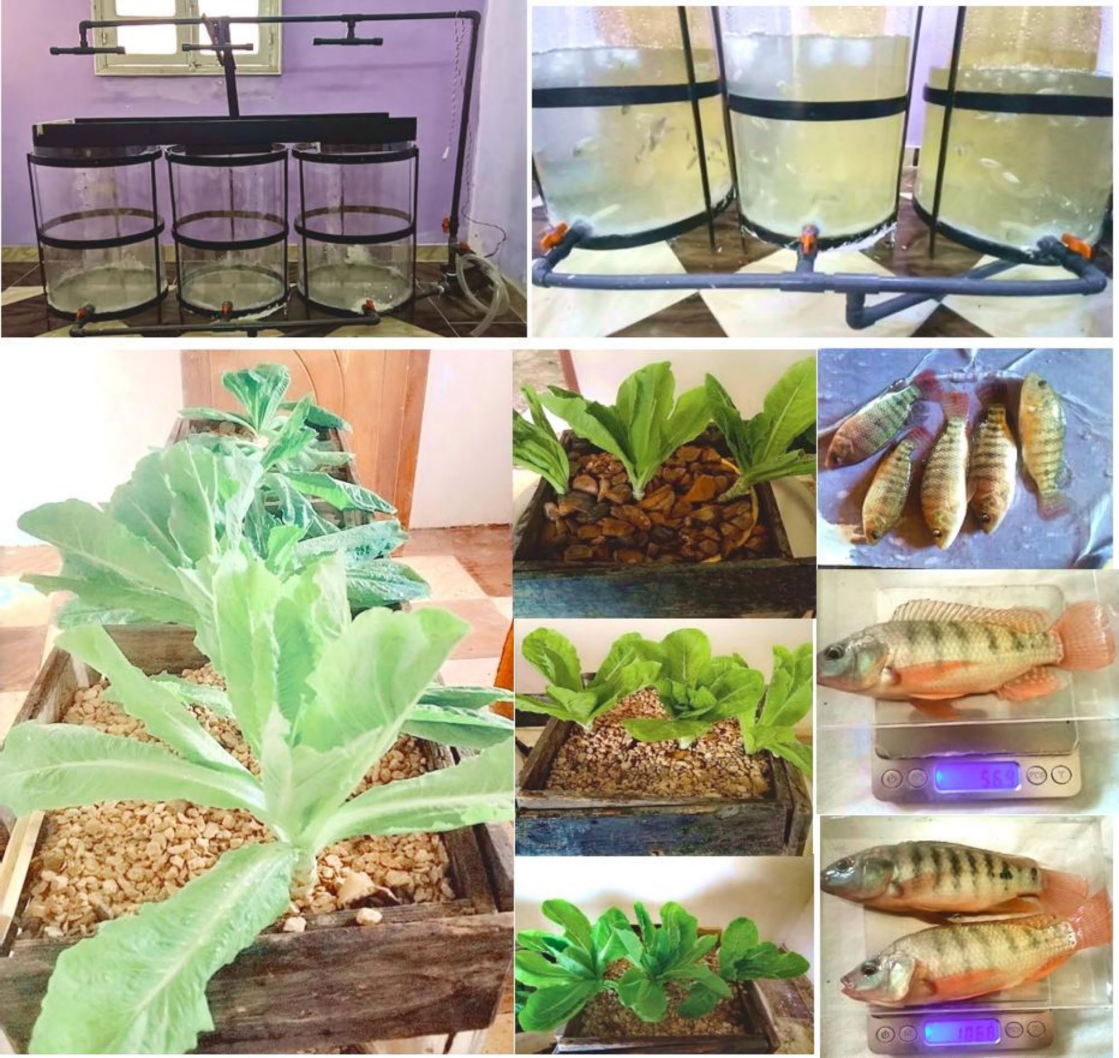
The photographs of the aquaponics system (fish tanks and wooden boxes for lettuce plants) and fish samples.

The experimental aquaponic system was designed, manufactured, and implemented in the laboratory of the Al Gamaleya Center (31°51′56.7 N and 31°10′44.2 E), Dakahlia Governorate, Egypt. The experimental work of the present study was carried out during the period from September 2022 to April 2023. Four experimental units were designed and constructed. Each unit consists of three systems of aquaponics as small-scale as shown in Fig. 3. Each system consists of two parts: the first part was a cylindrical fish tank; the height of the water in each of them was 60 cm, and therefore the volume of water in the tank was 108.57 L. Each tank holds 10 fish fries. Tilapia fry were purchased from Hassan Sobh Farm in Dakahlia, Dakahlia Governorate. The average weight of the fish at the beginning of the experiment was 25 ± 5 g. The second part is a wooden box with soil from rocks of different sizes. The volume of growth media was 0.00961 m³. Lettuce plants are planted in it, and in each box, there are 3 plants. The fish were fed twice daily at a rate of 4–7% of the total weight of the fish in each tank according to the weight of the fish in grams. 10. An amount of water from the tank is withdrawn every hour for a quarter of an hour using a pump that discharges 34 L/min in order to recycle the water and then filter it so that the plant can benefit from it.

### Parameters calculations

#### Fish growth performance in aquaponic system

The average individual weight (g) of the fish was gauged at the start and end of the week. At the end of each week, the weight gain (g), growth rate (g/day), feed conversion ratio, feed efficiency ratio, and survival rate (percentage) were calculated as^22,23^.

1 $${\text{Final body weight }}\left( {{\text{FBW}}} \right)=\,\frac{{Total~weight~of~fish~in~each~tank~\left( g \right)~}}{{Number~of~fish~in~each~tank}}$$

2 $${\text{Weight gain }}\left( {{\text{g}}/{\text{week}}} \right)\,=\,Final{\text{ }}weight - Initial{\text{ }}weight$$

3 $${\text{Weight gain }}\left( {{\text{WG}}\% } \right)\,=\,\frac{{Average~final~weight~\left( g \right)~ - ~Average~initial~weight~\left( g \right)}}{{Average~initial~weight~\left( g \right)}} \times {\text{1}}00$$

4 $${\text{Specific growth rate }}\left( {{\text{SGR}}/{\text{week}}} \right)=\frac{{Ln~final~weight~\left( g \right) - ~Ln~initial~wgight~\left( g \right)}}{{Week}} \times {\text{1}}00$$

5 $${\text{Feed conversion ratio }}\left( {{\text{FCR}}/{\text{week}}} \right)=\,\frac{{Feed~intake~\left( g \right)}}{{Weight~gain~\left( g \right)}}$$

6 $${\text{Feed efficiency ratio }}\left( {{\text{FER}}/{\text{week}}} \right)=\frac{{Weight~gain~\left( g \right)}}{{Feed~fed~\left( g \right)}} \times {\text{1}}00$$

### Statistical analysis

The data were statistically analyzed using SPSS^®^ Statistics 25, and the arithmetic means of the measured and calculated values were calculated monthly over the days of the experiment, and their standard deviations were calculated. Multivariate analysis of variance (MANOVA) was used, which is a statistical technique used to analyze differences between two or more groups when there are multiple dependent variables. The primary goal of MANOVA is to determine whether the means of the dependent variables differ significantly across groups while taking into account the interrelationships between the variables. The effect of fish growth environment (tanks), growing time, and their interaction as independent variables on water quality indicators was studied.

## Results and discussion

In order to study and calibrate the performance of the aquaponics system, lettuce plants were planted to utilize the system water, filter it, supply the system with oxygen, and measure water quality indicators. Then, different indicators of fish growth were calculated. Different growth indicators of lettuce plants were also measured.

### Fish growth performance of the system aquaponic

**Fig. 4:**
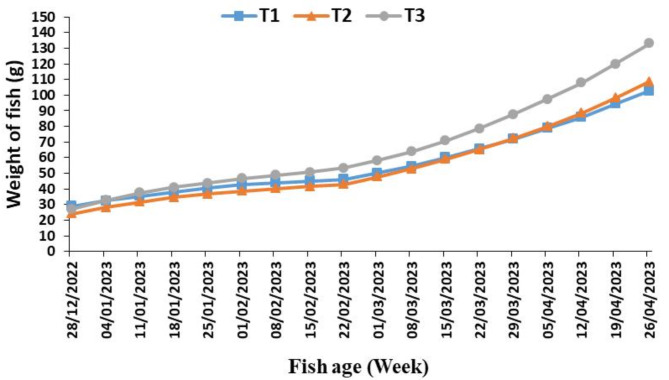
The weekly average weight of fish for three tanks (T_1_, T_2_ and T_3_) of the aquaponic system during the experiment period.

Growth parameters of fish in the aquaponics system, such as weight gain of fish (WG, g), feed conversion rate (FCR), and feed efficiency ratio (FER%), were calculated by measuring the weight of the fish weekly and the amount of feed provided accordingly. Figure 4 shows the weekly average weight of fish in the three tanks (T_1_, T_2,_ and T_3_) of the hydroponic system. The highest weight of fish throughout the experiment was in the third pond, followed by the first and second ponds, respectively. However, the highest average weight at the end of the experiment was for fish in the third pond, followed by the second pond, then the first pond, respectively, and the values were 133.1, 108.7, and 102.7 g. These results led to optimum environmental conditions (agreement with^22,23^).

**Fig. 5:**
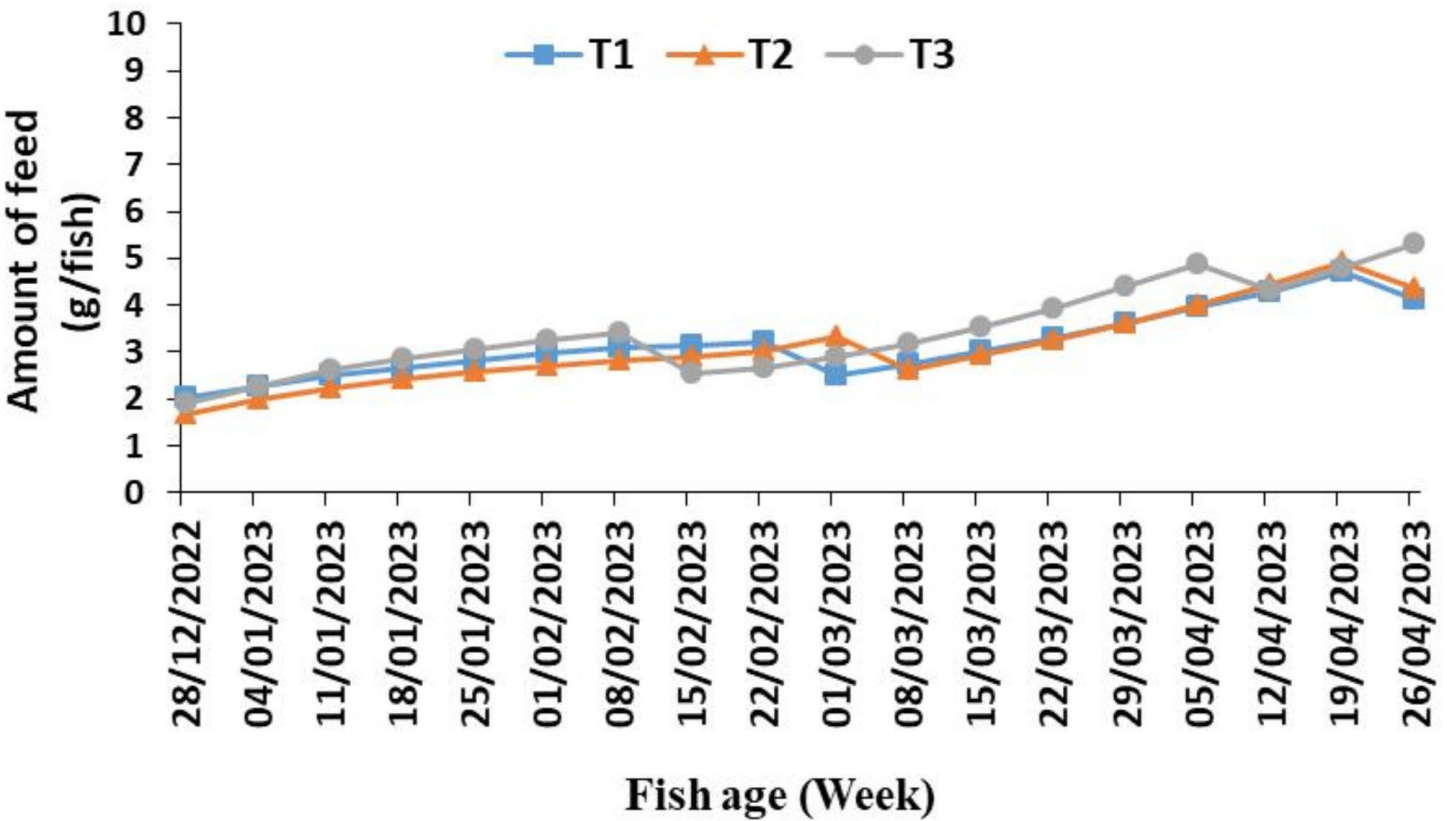
The weekly average amount of feed added to each fish to feed in three tanks (T_1_, T_2_ and T_3_) of the aquaponic system during the experiment period.

Figure 5 shows the weekly average amount of feed added to each fish to feed it in grams in the three tanks. We notice that the weekly average of the amounts of feed added to feed the fish increases from the beginning of the experiment until it reaches its maximum at the end of the experiment due to the increase in temperature and thus the increase in metabolism and weight gain of the fish. The highest amounts of feed consumed by the fish in the third tank over the days of the experiment were from 1.89 to 5.32 g (agreed with^24^).

**Fig. 6:**
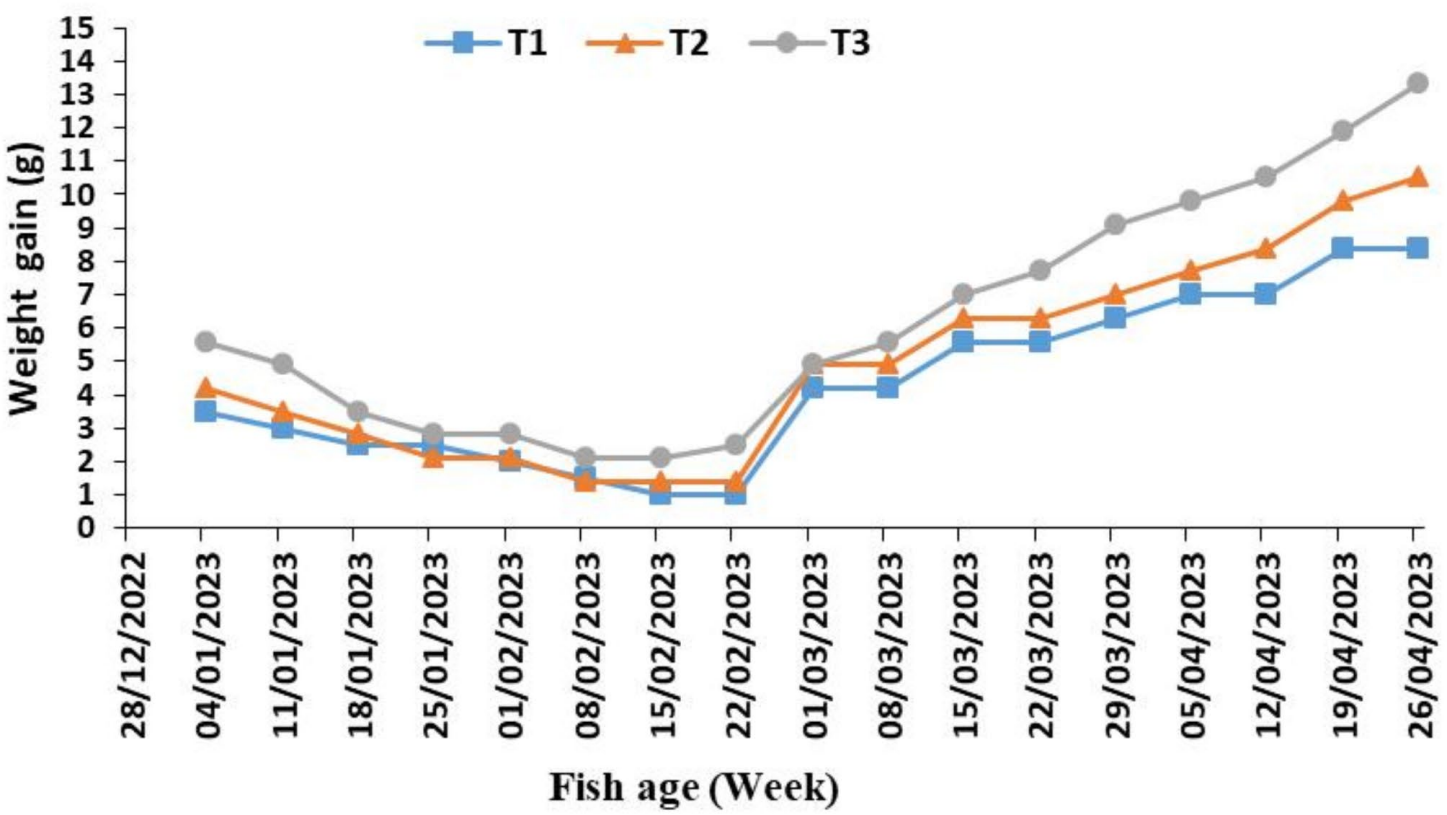
The weekly average weight gain of fish in three tanks (T_1_, T_2_ and T_3_) of the aquaponic system during the experiment period.

Figure 6 shows the weekly average weight gain of fish (WG, gm) in the three tanks. Weight gain from the beginning of the experiment to the end of February was at a decreasing rate due to lower temperatures during this period, then it started to increase at an increasing rate towards the end of the experiment. Tank 3 had the highest gain rates during the experimental period, ranging from 2.1 to 13.3 g.

**Fig. 7:**
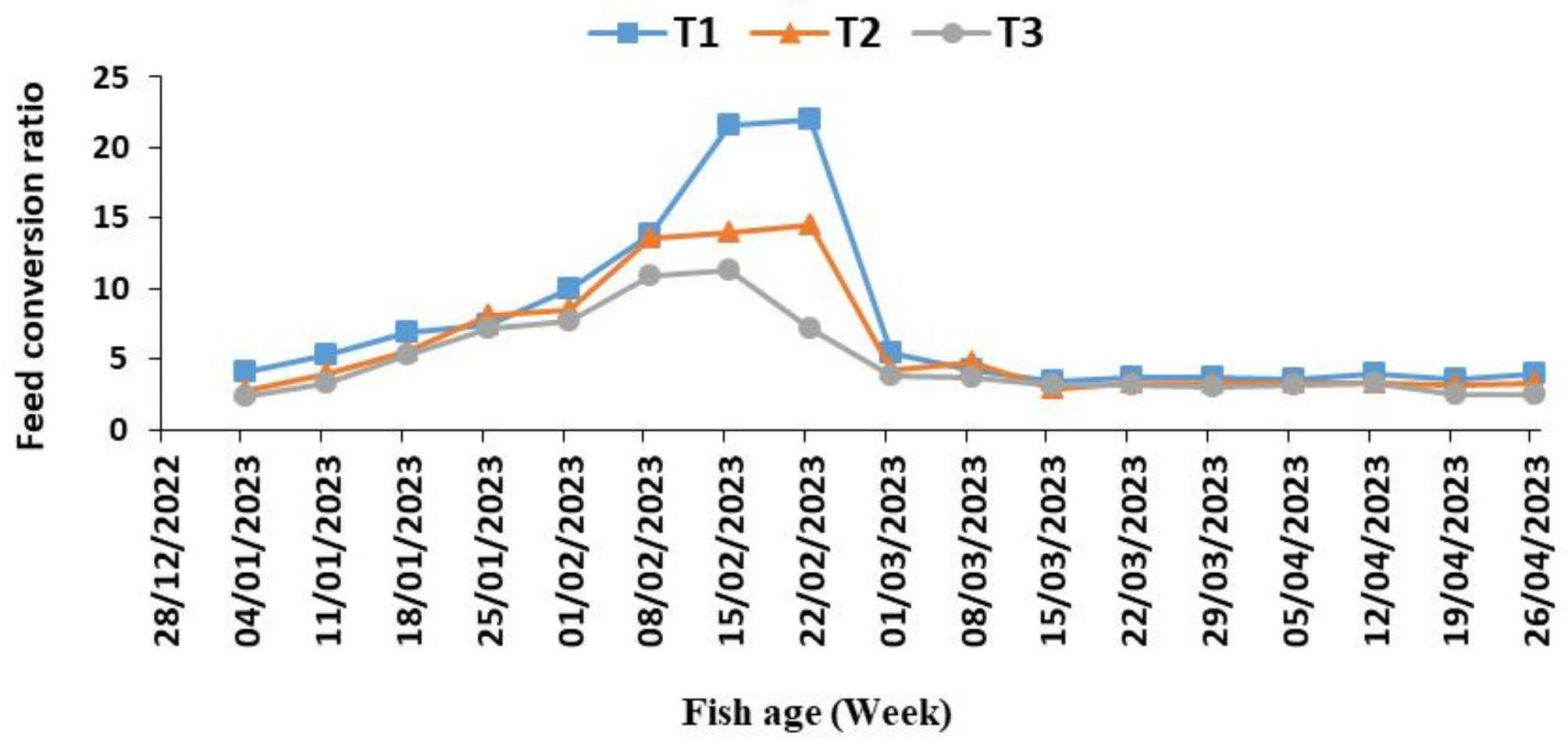
The weekly average feed conversion rate (FCR) of fish in three tanks (T1, T2 and T3) of the aquaponic system during the experiment period.

Figure 7 shows the weekly average feed conversion rate (FCR) for each fish in the three tanks. We note that the weekly average FCR of the feed added to the fish is lower at the beginning and end of the experiment due to the high temperature and thus the increased metabolism and weight gain of the fish. It increases in the middle of the experiment until it reaches its maximum as a result of the low temperatures of the experiment, so the metabolism was lower by the constant amount of feed as a percentage of weight (agreement with^25^).

**Fig. 8:**
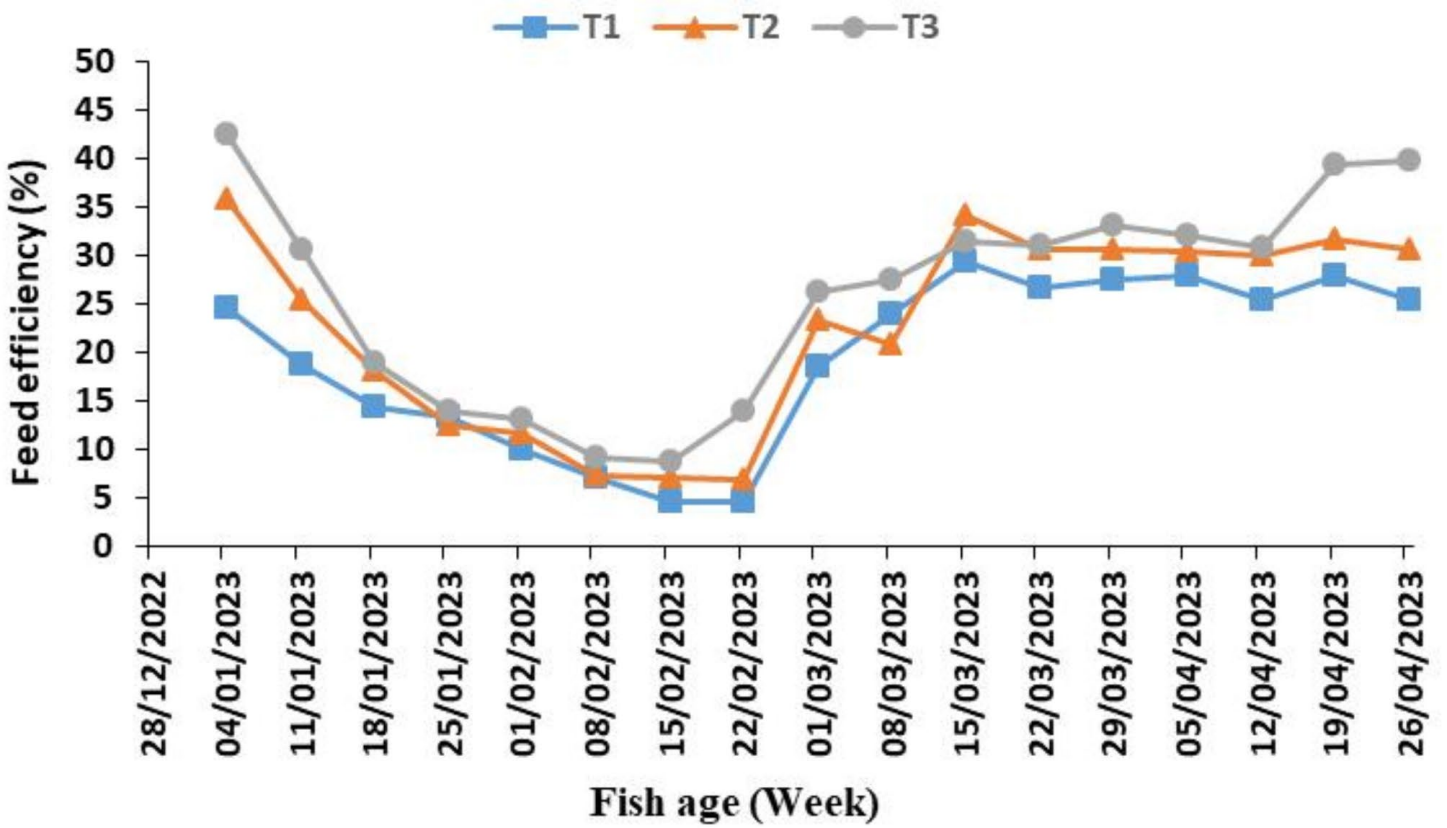
The weekly average feed efficiency (FER%) of fish in three tanks (T_1_, T_2_ and T_3_) of the aquaponic system during the experiment period.

Figure 8 shows the weekly average of feed efficiency (FER%) for the three tanks. It was highest at the beginning and end of the experiment and was lower in the middle of the experiment. The best treatment was for the third tank throughout the experiment. These results were in agreement with^18^.

### Effect of growth media on water quality parameters of the system aquaponic

Water quality parameters were measured in the three tanks. The results showed that these waters (dissolved oxygen, ammonia, pH, temperature, dissolved solids, and electrical conductivity) were measured in the aquaponics system for Nile tilapia farming and are shown in Table 2. The weekly average of dissolved oxygen values ranged from 5.80 to 8.00 ppm in all stages of the experiment, with the lowest value in the first tank and the highest value in the third tank (agreed with^10.8^). The weekly average of ammonia values ranged from 0.02 to 0.24 ppm, with the highest value in the first tank (according to^12,13^). The weekly average of water temperature ranged from 15 to 24 °C, as it did not change in the three tanks. The weekly average of pH values ranged from 6.8 to 8.3, with the lowest value in the second tank and the highest value in the first tank (according to^7,8^). The weekly average of total dissolved solids values ranged from 292 to 475 ppm, and the weekly average of electrical conductivity values ranged from 584 to 985µS/cm. The current results also showed that the levels recorded in the three tanks for water quality indicators do not exceed the permissible levels required for fish farming and production, but the third tank was the best in general.

**Table 2:** Highest and lowest weekly average measured values of water quality indicators in an aquaponics system in three tanks.

Measurements	Max			Min			Average ± SD		
	T_1_	T_2_	T_3_	T_1_	T_2_	T_3_	T_1_	T_2_	T_3_
DO, ppm	7.2	7.5	8	5.8	6	6.8	6.65 ± 0.47	6.86 ± 0.46	7.30 ± 0.46
NH_3_, ppm	0.24	0.17	0.14	0.02	0.02	0.02	0.12 ± 0.06	0.09 ± 0.04	0.08 ± 0.04
TEMP, °C	24	24	24	15	15	15	18.67 ± 3.24	18.67 ± 3.24	18.67 ± 3.24
pH	8.3	8	7.5	7	6.8	6.9	7.40 ± 0.39	7.26 ± 0.36	7.12 ± 0.15
TDS, ppm	475	466	365	292	292	292	397.00 ± 56.6	391.83 ± 55.46	329.00 ± 19.05
EC, µs/cm	985	900	830	584	584	584	810.00 ± 116.71	764.67 ± 83.73	704.67 ± 81.02

### Effect of growth media and water quality on plant quality parameters of the system aquaponic

**Fig. 9:**
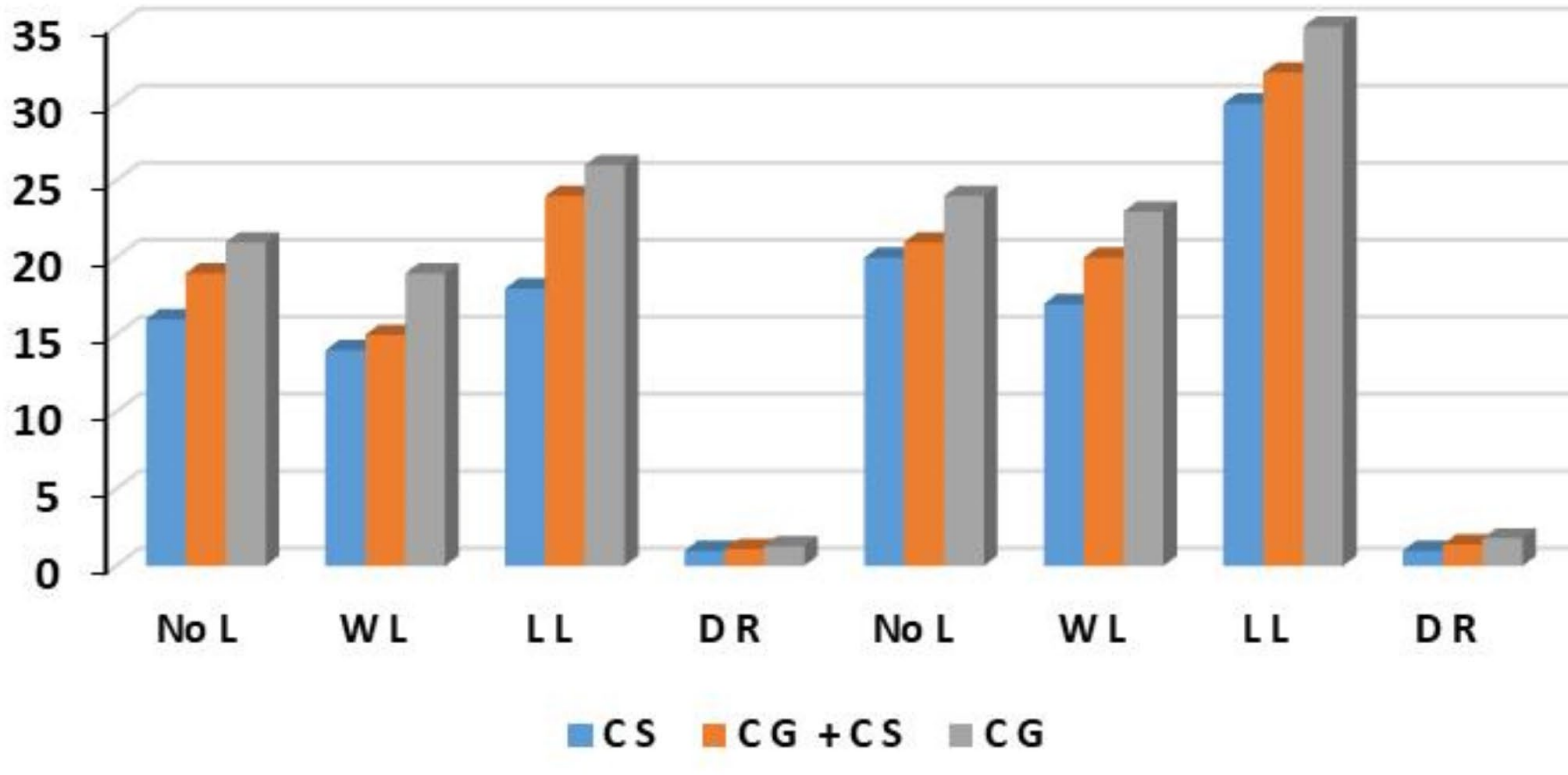
Final values at the end of the two cycles for all lettuce plant parameters (number of leaves (No L), leaf width (L W), leaf length (LL) and root diameter (RD)) in the three boxes with the three different media in the aquaponics system.

Plant quality indicators were measured weekly in the three boxes with the three different media, such as Crushed Stone (CS); the second box was Crushed Gravel + Crushed Stone (CG + CS); and the third box was Crushed Gravel (CG). Two cycles of lettuce were planted over the course of the experiment. The results showed that these indicators (number of leaves (No L), leaf width (L W), leaf length (LL), and root diameter (RD)) were measured in the aquaponics system for Nile tilapia farming. The final values at the end of the two crops for all indicators ranged as shown in Fig. 9. The best treatment in the measured values of plant quality indicators was the third box with crushed gravel medium in both cultivation cycles. These results agreed with those obtained by^26^.

### Statistical analysis of the system aquaponic

Table 3 shows the results of the statistical analysis of the MANOVA model to test the effect of time and tank as an independent variable for the accompanying variables, which are ammonia (NH_₃_), dissolved oxygen (DO), and interaction between them. The most important results of this analysis were the significant p-values for the indicators NH_3_ and DO with T and t. The interaction T*t was not significant for DO and significant for NH_3_. The R² values were 0.914 and 0.873, respectively, for the growth indicators as follows: NH_₃_ and DO, respectively.

**Table 3:** MANOVA model to examine the effect of time and tank on ammonia (NH_3_) and dissolved oxygen (DO).

Parameter	Tanks			T	t
	T_1_	T_2_	T_3_	*P*-Value	
	M ± SD	M ± SD	M ± SD		
NH_3_	T * t → ≤ 0.05			**R**^**2**^ **= 0.914**	
Jan.	0.05 ± 0.02	0.04 ± 0.01	0.03 ± 0.01	≤ 0.05	≤ 0.05
Feb.	0.10 ± 0.01	0.07 ± 0.01	0.06 ± 0.01		
Mar.	0.13 ± 0.03	0.10 ± 0.02	0.09 ± 0.01		
Apr.	0.21 ± 0.03	0.15 ± 0.02	0.13 ± 0.01		
DO	T * t → ≥ 0.05			**R**^**2**^ **= 0.873**	
Jan.	7.02 ± 0.04	7.26 ± 0.21	7.76 ± 0.43	≤ 0.05	≤ 0.05
Feb.	7.03 ± 0.21	7.18 ± 0.21	7.55 ± 0.17		
Mar.	6.56 ± 0.21	6.72 ± 0.20	7.04 ± 0.15		
Apr.	5.93 ± 0.15	6.20 ± 0.22	6.80 ± 0.00		
	NH_3_ *DO			T*t	
NH_3_				≤ 0.05	
DO				≥ 0.05	

Significant values are in bold.

## Conclusion

The overall results of this experiment indicated that the aquaponics system used is water-saving as the water is recycled and filtered through irrigation of lettuce and fertilized with fish waste. The third treatment with the medium had the best performance throughout the experiment in terms of fish growth rate, water quality indicators, and plant growth indicators. It provided approximately 50% of the water required for the aquaponic system compared to other units. These hydroponic systems have shown potential in alternative agriculture applications and can be promoted as an indoor or outdoor cultivation system. The advantages of these hydroponic systems over conventional cultivation systems are higher density growth, lower water usage, year-round crop production, pest and disease control, and weed control. However, further research is needed on the feasibility of each system. Thus, increased food safety of products produced through hydroponics is ensured. Studies on the quality and risks of consumption of products produced in hydroponic systems should be conducted more comprehensively.

## Data Availability

All data obtained or analysed in this study are included in the publication. Additionally, the analyses can be requested from the corresponding author. No datasets were generated or analysed during the current study.
